# Effects of the Urban Environment on Oxidative Stress in Early Life: Insights from a Cross-fostering Experiment

**DOI:** 10.1093/icb/icy099

**Published:** 2018-07-20

**Authors:** Pablo Salmón, Hannah Watson, Andreas Nord, Caroline Isaksson

**Affiliations:** 1 Section for Evolutionary Ecology, Department of Biology, Lund University, Lund, SE-223 62, Sweden; 2 Institute of Biodiversity, Animal Health and Comparative Medicine, College of Medical, Veterinary and Life Sciences, University of Glasgow, Glasgow, G12 8QQ, UK

## Abstract

As urban areas expand rapidly worldwide, wildlife is exposed to a wide range of novel environmental stressors, such as increased air pollution and artificial light at night. Birds in highly polluted and/or urbanized habitats have been found to have increased antioxidant protection, which is likely important to avoid accumulation of oxidative damage, which can have negative fitness consequences. Yet, the current knowledge about the ontogeny of antioxidant protection in urban areas is limited; i.e., is the capacity to up-regulate the antioxidant defences already established during pre-natal development, or does it manifest itself during post-natal development? We cross-fostered great tit (*Parus major*) nestlings within and between urban and rural habitats, to determine if oxidative stress (measured as non-enzymatic total antioxidant capacity, superoxide dismutase (SOD), and plasma lipid peroxidation) is affected by habitat of origin and/or by habitat of rearing. The results demonstrate that being reared in the urban environment triggers an increase in SOD (an intracellular, enzymatic antioxidant) independent of natal habitat. Oxidative damage increased with hatching date in urban-reared nestlings, but there was little seasonal change in rural-reared nestlings. Total antioxidant capacity was neither affected by habitat of rearing or habitat of origin, but we observed a decline with hatching date in both rearing habitats. Taken together, our results support the growing evidence that the urban environment induces a direct plastic adjustment in antioxidant protection, but that up-regulation is not sufficient to avoid increased oxidative damage in late-hatched broods. Future studies should explore the underlying causes for this effect in late-hatched broods and whether it has any negative long-term implications, both at the individual- and the population level.

## Introduction

Urbanization is one of the largest current threats to global biodiversity, and its expansion rate will increase in the future, as 85% of the human population is predicted to live in cities by 2050 compared with 50% in 2008 (Seto et al. 2011, 2012; United Nations 2016). Thus, it is important to investigate species’ resistance and resilience to such rapid changes in order to understand present and future threats from urbanization. Physiological adaptation may be crucial in determining species’ responses to environmental degradation (Chown and Gaston 2008) and may underlie the ability of species and individuals to successfully exploit urban environments (e.g., Partecke et al. 2006; Møller 2009; Møller et al. 2010; Bonier 2012; Dominoni et al. 2013; Bailly et al. 2016).

Oxidative stress is regarded as an important mediator of life-history trade-offs due to the need to balance metabolic efficiency and generation of pro-oxidants—a by-product of aerobic respiration (Monaghan et al. 2009). If increased levels of reactive oxygen species/reactive nitrogen species (ROS/RNS) cannot be balanced by concomitant increases in antioxidant defences, oxidative stress occurs. The resultant accumulation of oxidative damage can have negative consequences for individual fitness, e.g., survival (Bize et al. 2008; Noguera et al. 2011; Losdat et al. 2013; Herborn et al. 2016; Crommenacker et al. 2017). Many so-called “urban stressors,” such as traffic-related pollution, poor diet quality, and exposure to novel pathogens can directly or indirectly affect an individual’s oxidative status (reviewed by Isaksson 2015). This supports the idea that the regulation of oxidative stress, mostly via the antioxidant machinery (either dietary or endogenously derived), could be one of the key mechanisms determining the capacity to cope, or failure to cope, with living in an urban environment.

The predictions regarding oxidative stress physiology in relation to urbanization may seem straightforward, i.e., the higher the urbanization intensity, the greater the upregulation of antioxidant responses to maintain homeostasis. If antioxidant defences are overwhelmed, oxidative damage to lipids, proteins, and DNA will occur. However, empirical studies comparing urban and rural populations of birds are far from conclusive (e.g., Isaksson et al. 2009, 2017; Giraudeau and McGraw 2014; Herrera-Dueñas et al. 2017). It appears that both the antioxidant response and the accumulation of oxidative damage are not only highly marker-specific, but also species-, life-stage- (early-life or adulthood), and context-specific (type of pollutants and time of the year) (e.g., Isaksson et al. 2017; Salmón et al. 2018). In addition, it is well known that maternal phenotype or experience can influence the offspring phenotype (Mousseau and Fox 1998; Marshall and Uller 2007; Groothuis and Schwabl 2008), and these effects can be an effective mean of buffering offspring from environmental stressors (Agrawal et al. 1999). Such maternal effects could shape the oxidative physiology of urban offspring to match their future environment (De Coster et al. 2012; Giordano et al. 2015). Yet, few studies have tried to experimentally disentangle how the urban environment affects the ontogeny of such responses (but see Isaksson et al. 2006; Partecke et al. 2006; Costantini et al. 2014). A common garden study, in which urban and rural European blackbirds (*Turdus merula*) were brought into captivity at 5–11 days, showed that the oxidative stress response of urban individuals to chronic stress (repeated immune and disturbance stressors) was distinct from rural birds even after a year in captivity (Costantini et al. 2014). This demonstrates the potential for long-lasting effects of exposure to the urban environment. However, there is evidence that the phenotypic response to urbanization and its evolutionary potential is unlikely to be uniform across species, populations, and latitudes (Møller 2009), and further studies are needed to better understand the effects of urban habitats during early-life.

In the present study, we performed a cross-fostering experiment within and between urban and rural habitats, shortly after hatching, using great tit (*Parus major* L.) nestlings. Previous research in this species has shown multiple phenotypic differences between urban and rural populations with possible links to fitness, such as immune response (Bailly et al. 2016), behavioral ecotypes (Charmantier et al. 2017; Senar et al. 2017), plumage coloration (Isaksson et al. 2005, 2006), breeding performance (Sprau et al. 2017), and oxidative stress physiology (Isaksson et al. 2005, 2017). However, little is known about whether such phenotypic differences manifest during pre-natal development or if they are determined by the rearing environment during post-natal development. Our objectives were (i) to examine if there are differences in some oxidative stress components during early-life between urban and rural individuals, and (ii) in case of differences, disentangle if they are driven by the pre- or post-hatching habitat.

## Materials and methods

### Study areas and experimental design

The experiment was performed during the breeding season of 2013 (April–June) in an urban and a rural nest-box population of great tits, a common Eurasian passerine bird. The urban study area was located in the city of Malmö, Sweden’s third largest city with approximately 310,000 inhabitants. The rural population was located in a forest 37 km ENE of Malmö. Nest-boxes in the urban habitat were situated in three city parks, comprising a mix of deciduous trees, managed grassland, and hard surfaces. The rural study area was part of a continuously forested, pine-dominated, area (see Supplementary Information in Salmón et al. [2016] for further details).

All nest-boxes were visited weekly in the beginning of the breeding season to determine the day of the first egg (back-calculated assuming one egg was laid each day) and clutch size. When nestlings were 2 days (hatching day = 0), we cross-fostered half of the nestlings from a brood (median ± SD: 3 ± 0.6) in the urban study area with the same number of nestlings from a nest of identical age and similar brood size (±1 nestling) from the rural study area (*n *=* *16 nest pairs). In addition, we cross-fostered nestlings within each study area to assess the effect of the cross-fostering itself (urban: *n* = 8 nest pairs; rural: *n* = 10 nest pairs). When brood size differed within pairs, we swapped the number of nestlings corresponding to half the number of the smaller brood, and when (if) the brood size had an uneven number we swapped the lowest number of nestlings (e.g., three nestlings when the brood size was 7). Before manipulation, nestlings were ranked according to body mass, and every other nestling was chosen for the swap (e.g., nestling 1, 3, 5 or 2, 4, 6; starting from the lightest and second-to-lightest chick on alternate swaps). Cross-fostered nestlings were individually marked by clipping the outermost tips from the claws. There were no differences in body mass between cross-fostered and non-cross-fostered nestlings, or between habitats at time of cross-fostering (linear mixed-effect model [LMM]: *F*_1,__363.82_ = 0.55; *P *=* *0.460 and *F*_1,__286.06_ = 0.54; *P *=* *0.460, respectively). Breeding start (median ± SD: urban, 8 ± 4; rural, 8 ± 3, days from the first of May) did not differ between habitats (*F*_1,__58_ =1.86; *P *=* *0.177).

When nestlings were 15 days, morphometric measures (body mass, wing and tarsus length) were recorded, and a blood sample (100 µL) was collected from the jugular vein into a heparinized tube and immediately stored on ice. Samples were centrifuged for 10 min at 1800 rpm 0–1 h later to separate plasma from red blood cells (RBCs). The plasma and RBC were then snap-frozen in liquid nitrogen and, at the end of the field day, transferred to storage at −80°C until analyses.

### Molecular sexing

Molecular sexing of nestling was carried out using primers P2 and P8, following (Griffiths et al. 1998).

### Superoxide dismutase

Superoxide dismutase (SOD) was quantified using a colorimetric assay kit (Sigma–Aldrich, Stockholm, Sweden). RBCs were diluted and homogenized 1:1 with PBS, then diluted 1:3 with ddH_2_O and centrifuged for 14 min at 4°C at 10,000 × g. Ten microliters of the obtained supernatant was then further diluted (1:9) with the Dilution Buffer (provided in the kit); 20 µL of the diluted supernatant was used in the assay, according to the manufacturer’s protocol. Following addition of the enzyme working solution, the plate was shaken and the absorbance measured every minute for 15 min at 450 nm and at 40°C (i.e., slightly below the average active body temperature of birds; Prinzinger et al. [1991]). SOD activity (U mL^−1^) was calculated relative to a standard curve ranging from 50 to 1.56 U mL^−1^. SOD activity was corrected for the amount of protein present in the sample (mg mL^−1^) quantified by the Bradford method (Bradford 1976) and relative to a standard curve of bovine serum albumin (1.5–0.125 mg mL^−1^) at an absorbance of 595 nm. All samples were measured in duplicates and the repeatability was very high following Lessells and Boag (1987) (ICC = 0.97, 95% CI = 0.96–0.97, *F*_1,__304_ =4.90× 10^3^, *P < *0.001).

### Total antioxidant capacity

Total antioxidant capacity (AOX) of the plasma was measured using the ferric reducing antioxidant power (FRAP) assay, which gives the overall reducing potential, i.e., the non-enzymatic antioxidant potential, of the sample (Benzie and Strain 1996). Briefly, 5 µL plasma was diluted 1:8 with ddH_2_O and 20 µL of the diluted plasma sample was then incubated with 150 µL working solution (sodium acetate trihydrate + 2, 4, 6-Tris (2-pyr-idyl)-s-triazibe [TPTZ] +Iron [III] chloride hexahydrate [FeCl_3_⋅6H_2_O]; 10:1:1) for 20 min at room temperature. Immediately following incubation, the color generated from the reduction of Fe^3+^ (ferric) to Fe^2+^ (ferrous) was measured at 593 nm. Known Fe^2+^ concentrations (Iron [II] sulphate heptahydrate [${\text{FeSO}}_{4}^{-7}\cdot {\text{H}}_{2}\text{O}$]) were used as standard curve. All chemicals were purchased from Sigma–Aldrich (Stockholm, Sweden). Uric acid levels were measured in 5 µL of plasma using a commercial kit (SPINREACT, Sant Esteve d’en Bas, Spain) based on the uricase/peroxidase method. In both assays, all samples were run in duplicate with high repeatability (FRAP: ICC =0.93, 95% CI = 0.91–0.95, *F*_1,__259_ =4.00× 10^3^, *P < *0.001; Uric acid: ICC = 0.99, 95% CI = 0.99–0.99, *F*_1,__259_ =1.80× 10^4^, *P < *0.001). Up to 90% of the variation in avian plasma AOX can be due to the effect of uric acid (Cohen et al. 2007; Costantini 2011). To statistically control for uric acid levels on antioxidants, we used the residuals from a regression model with our antioxidant measure as the dependent variable and uric acid as the predictor as our measure of antioxidant capacity (hereafter AOX).

### Lipid peroxidation

Malondialdehyde (MDA) was first extracted from 10 to 15 µL of plasma following the protocol described in Eikenaar et al. (2016). Briefly, samples were vortexed with 50 µL buffer (1 mM *O*-(2,3,4,5,6-pentafluorobenzyl) hydroxylamine hydrochloride in 1.5 M sodium acetate buffer pH 5.0) and incubated at room temperature for 1 h (with vortexing at 30 min). To this, 300 µL of heptane with internal standard (1.57 fg µL^−1^ 1-bromo-3-fluorobenzene) was added. Following vortexing, the lower phase was carefully removed by pipette, leaving the upper phase containing MDA. Extracts subsequently went through two to three washing steps: 200 µL distilled water was added, followed by vortexing and removal of the lower phase. Residual water was removed by the addition of anhydrous sodium sulfate. Extracts were finally dried under nitrogen gas, leaving a final volume of 40–50 µL. MDA was then quantified by gas chromatography–mass spectrometry (GC/MS) using an Agilent 5975 MS coupled to an Agilent 6890 GC with a non-polar capillary column, HP-5MS (30 m, 0.25 mm id, df 0.25 µm; J&W Scientific, USA). The GC oven was programmed to 60°C for 1 min, followed by 15°C/min to 150°C, and then 10°C/min to 270°C, which was held for 5 min.

### Statistical analysis

All statistical analyses were conducted in R 3.2.4 (R Core team 2015). The effects of the habitats of origin and rearing on the different oxidative stress biomarkers were modeled using LMMs fitted with maximum likelihood methods and normal error structures using the lme4 package (Bates et al. 2015). The nest of origin (to account for genetic effects) and the nest of rearing (to account for the effects of the common environment) were included as random effects. Two sets of models were fitted: (i) between-habitat cross-fostering for testing the effect of habitat of rearing and origin; and (ii) within-habitat cross-fostering to test for the potential effect of the manipulation (cross-fostering) *per se* (see Supplementary Tables S1 and S2 in the Supplementary Material for details on the full and final models). The between-habitat model for each biomarker included habitat of origin (urban/rural), habitat of rearing (urban/rural), and sex as fixed factors, and body mass and hatching date as covariates. The original model also included all two-way interactions with habitat of rearing. Our ultimate objective was to evaluate the effect of the latter on our oxidative stress biomarkers; thus, we discarded the inclusion in the model of any interactions with habitat of origin in order to avoid overparameterization. For SOD and AOX, the laboratory assay plate was included as a random effect. The within-habitat model was similar, though it contained only a single habitat variable (since habitats of origin and rearing were the same), and the interaction between manipulation (cross-fostered or not cross-fostered) and habitat. MDA levels were log transformed to achieve normality.

Final models were derived by backward elimination of non-significant terms based on likelihood ratio tests until only significant (*P* < 0.05) variables remained. The final models were then refitted with restricted maximum likelihood (Zuur et al. 2009). Denominator degrees of freedom for fixed effects were calculated using the Satterthwaite approximation using the *lmerTest* package (Kuznetsova et al. 2017).

## Results

### Superoxide dismutase

**Fig. 1:**
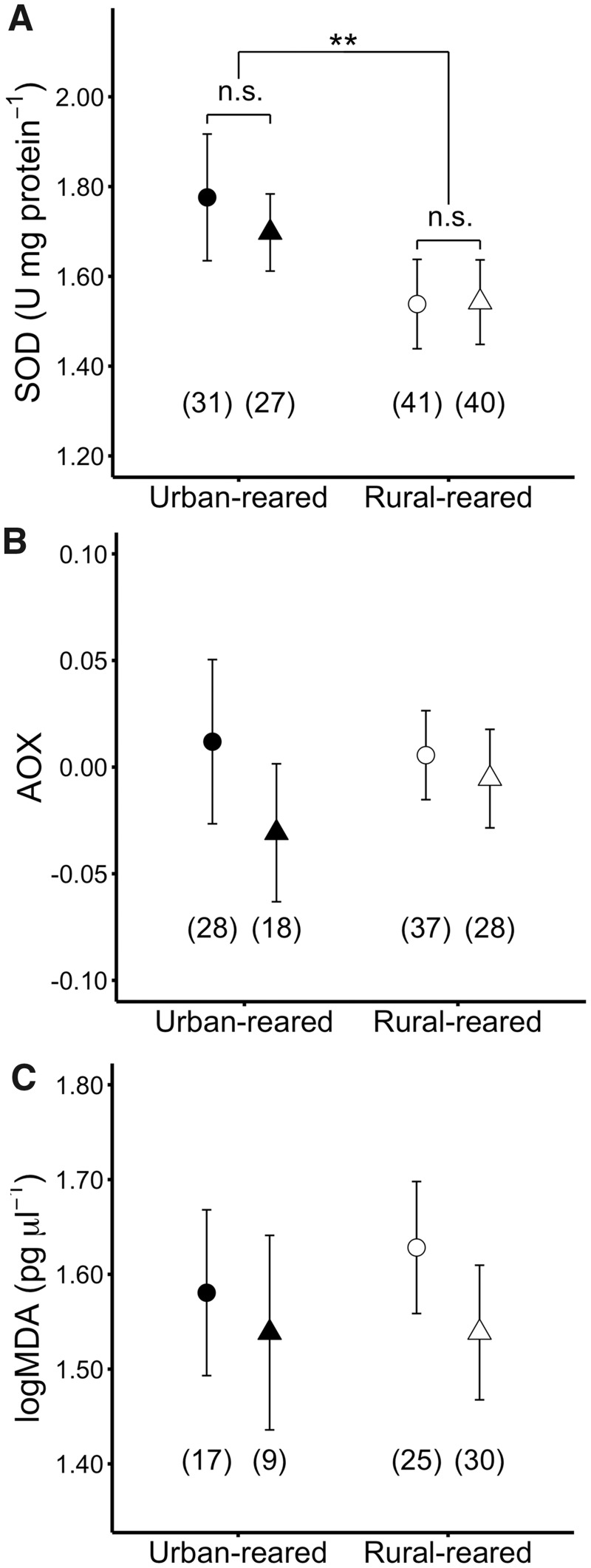
Mean±SE levels of: **A**) superoxide dismutase (SOD); **B**) plasma antioxidant capacity, (AOX; FRAP assay corrected for uric acid), and **C**) plasma lipid peroxidation (MDA) at 15 days in great tit nestlings in urban (black) and rural (white) rearing habitats. Circles denote non-cross-fostered nestlings, and triangles denote cross-fostered nestlings. Numbers below bars indicate number of nestlings.

The rearing environment significantly influenced SOD (*P* = 0.008, Fig. 1A), with the result that urban-reared nestlings showed higher SOD levels than rural-reared nestlings. This effect was independent of the habitat of origin (“rearing habitat ×habitat of origin”: *P* = 0.652, Fig. 1A). Neither body mass nor hatching date affected SOD levels (all *P* ≥ 0.344). The random effect of nest of rearing and nest of origin was non-significant (random variance_[nest of rearing__]_ =0.022, *P* = 0.160; random variance_[nest of origin__]_ =0.033, *P=*0.060; residual random variance =0.122). Sex did not affect SOD (*P* = 0.421).

### Antioxidant capacity

**Fig. 2:**
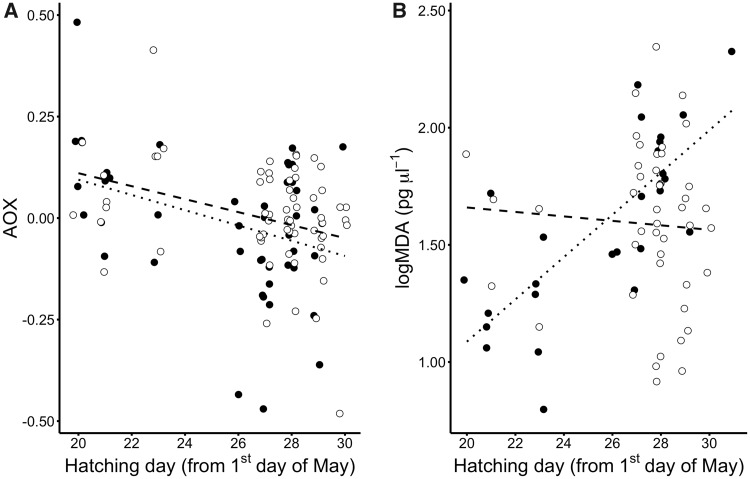
Relationship between **A**) plasma antioxidant capacity (AOX; FRAP assay corrected for uric acid), and hatching day (from 1 May); and **B**) plasma lipid peroxidation (MDA) and hatching day, in 15 days great tit nestlings reared in urban (black color and dotted line) and rural (white color and dashed line) populations.

AOX was not influenced by the rearing habitat (*P* = 0.331) or the habitat of origin (*P* = 0.469, Fig. 1B). However, nestling AOX levels were negatively related to hatching date (*P* =0.013, Fig. 2A), and this effect was independent of rearing habitat (“rearing habitat ×hatching date”: *P *=* *0.977). Sex and body mass did not affect AOX (*P *≥* *0.231). AOX varied between nests of rearing, but not between nests of origin (random variance_[nest of rearing__]_ =0.004, *P* = 0.040; random variance_[nest of origin__]_ =3.29× 10^−4^, *P=*0.860; residual random variance =0.014).

### Lipid peroxidation

MDA levels in plasma were significantly different between rearing habitats, but the effect was dependent on hatching date (i.e., “rearing habitat × hatching date”: *P*=0.003). Specifically, MDA increased over the season in urban-reared nestlings, but remained relatively stable in rural birds (Fig. 2B). The effect of rearing habitat on MDA levels was independent from the habitat of origin (“rearing habitat × habitat of origin”: *P* = 0.460). Neither body mass nor sex had an effect on MDA levels (*P *≥* *0.264). This was also the case for the random effects of nest of rearing and nest of origin (*P *>* *0.400; random variance_[nest of rearing__]_ =0.008; random variance_[nest of origin__]_ =0.000; residual random variance =0.009).

### Within-habitat model

For all oxidative stress markers, the within-habitat models showed that the manipulation *per se* (cross-fostering) did not affect the measured traits (“rearing habitat ×manipulation”: all *P *≥* *0.297; main effect of “manipulation”: all *P *≥* *0.248).

## Discussion

Our results demonstrate that, regardless of origin, being reared in an urban habitat elicits a direct physiological response to regulate oxidative stress in response to this environment. We found no evidence for a significant effect of habitat or nest of origin on any marker, indicating that the observed physiological changes in SOD (an antioxidant enzyme) and lipid peroxidation (a marker of oxidative damage) in urban-reared nestlings can be directly attributed to exposure to the urban environment during post-hatching development.

SOD is a key intracellular enzyme that scavenges superoxide radicals that are generated through leakage from the mitochondrial electron transport chain during metabolism (Halliwell and Gutteridge 2015). Although it is not possible to determine the urban factor(s) driving the upregulation of SOD in urban-reared nestlings, higher SOD levels are likely a response to increased ROS exposure (Sylvie et al. 2012). Growth is a demanding life stage characterized by elevated ROS production (Smith et al. 2016), and dietary constraints during this stage can explain a large part of the variation in the synthesis and levels of antioxidants (Li et al. 2014; Giordano et al. 2015; Noguera et al. 2015). There is evidence that urban environments might constrain nestling growth as a result of dietary restrictions (Pollock et al. 2017); yet, this potential diet difference is not reflected in our plasma antioxidant measurement (AOX), which includes dietary as well as endogenously synthesized (non-enzymatic) antioxidants circulating in the plasma (Costantini 2011). While non-enzymatic plasma antioxidants are affected by the systemic physiological and nutritional state, SOD, which is measured in RBCs, reflects the more tightly regulated environment within the cell. The absence of a habitat effect on non-enzymatic AOX could be explained if the physiological and nutritional state are more strongly affected by for example season rather than habitat type. With a limited blood volume, it was not possible to measure additional cellular antioxidants (e.g., catalase, glutathione, glutathione reductase). Thus, we cannot conclude if the urban upregulation of SOD is in response to a change in another cellular antioxidant, and thus an indirect effect of urbanization, rather than a direct effect of increased ROS (Isaksson et al. 2011).

Interestingly, the lack of an effect of the urban environment on nestling AOX contrasts with our recent studies in adult great tits, and three other passerine species, where antioxidant capacity was positively correlated with the intensity of urbanization (Salmón et al. 2018). Although that study was conducted during winter, the observed differences in AOX levels between adulthood and early life could be the result of post-fledging selective disappearance of individuals with low AOX in our urban population, which we have previously shown in relation to telomere length (Salmón et al. 2017). Alternatively, the immediate rearing environment of the nestlings, i.e., the enclosed nest-box, could have buffered the exposure of certain types of urban stressors, such as artificial light at night (Raap et al. 2018), such that urbanization effects on AOX are not readily observed before fledging.

Despite the overall upregulation of SOD in urban-reared nestlings, there was a steady increase in lipid peroxidation over the season in the urban environment, whereas SOD levels in rural-reared nestlings remained largely stable. Meanwhile, the seasonal decline in AOX did not differ between rearing habitats. Thus, hatching date strongly influenced the overall oxidative stress physiology of nestling great tits and more so in the urban environment. The seasonal trends in AOX (both environments) and MDA (urban environment only) could be mediated by seasonal changes in parental quality and/or food quality and availability. Breeding start is an important component of fitness in birds, and early broods are often laid by pairs with more experience and/or higher quality territories (e.g., Perrins 1965; Svensson and Nilsson 1995). Thus, in our study, the early broods may have been reared in high-quality territories independent of habitat type. Diet composition and quantity could become less favorable with the progression of the season, with a resultant decline in the availability of dietary antioxidants (Arnold et al. 2010). In a previous study of the same individuals, we showed that early broods were in better body condition (Salmón et al. 2016), which supports the idea that diet quantity and/or quality are higher early in the season. This is likely directly linked to the seasonal differences in antioxidant capacity observed in this study.

In wild vertebrate populations, previous cross-fostering experiments have shown that variation in ROS production and oxidative damage is explained by common origin (i.e., family; Costantini and Dell'Omo 2006; Olsson et al. 2008; but see Losdat et al. 2014). Yet, in the present study, none of the analyzed oxidative stress markers showed such effects (i.e., there was no significant variation explained by nest of origin), nor effects of the common rearing environment (i.e., nest of rearing; except for AOX). Age or developmental stage can affect the heritability and the additive genetic variance estimates of some traits (Charmantier et al. 2006), including the resistance to oxidative stress during postnatal development (Kim et al. 2010); it is therefore possible that the effect of the nest of origin in the expression of the studied oxidative stress markers could arise later in life. In addition, at the time of cross-fostering at 2 days, nestlings might have had access to antioxidants derived from the yolk or early diet (Surai 2002). Any such maternal effects in combination with the environmental rearing conditions might have overridden the detection of any variation in oxidative stress physiology explained by the nest of rearing.

In conclusion, our results show that urban rearing conditions during post-hatching development have a larger impact than habitat and nest of origin experienced during early development. Regardless of the underlying causal factors, increased oxidative stress can be a constraint and a cost during development (Smith et al. 2016), with long-term fitness effects (Metcalfe and Monaghan 2001). The present study suggests that the urban environment imposes a physiological challenge during post-hatching development in great tits. This is further corroborated by previous results demonstrating accelerated telomere attrition and lower survival probability for a given telomere length in urban-reared nestlings in the same populations (Salmón et al. 2016, 2017). However, we should be cautious when extrapolating the observed differences to other urban populations, due to regional variation in numerous biotic and abiotic factors (reviewed by Isaksson et al. 2018). Thus, we need more replicated studies in multiple cities to fully understand the consequences of developing within an urban habitat and its potential impacts on fitness.

## Supplementary Material

Supplementary Data
