# Supplementary material for: Effects of the Urban Environment on Oxidative Stress in Early Life: Insights from a Cross-fostering Experiment
Source: Integr Comp Biol. 2018 Jul 20;58(5):986–94. doi: 10.1093/icb/icy099 (PMC6204991; doi:10.1093/icb/icy099)
Supplement: Supplementary Data [file icy099_supp.docx]

### Supplementary Data

Effects of the urban environment on oxidative stress in early life: insights from a cross-fostering experiment

Pablo Salmón^1+^, Hannah Watson ^1^, Andreas Nord ^1^ and Caroline Isaksson ^1*^

^1^ Section for Evolutionary Ecology, Department of Biology, Lund University, Lund, Sweden

^+^ Present address: Institute of Biodiversity, Animal Health & Comparative Medicine, College of Medical, Veterinary & Life Sciences, University of Glasgow, Glasgow, UK

* Corresponding author: Caroline.Isaksson@biol.lu.se

Table S1. Linear mixed models of three oxidative stress biomarkers in 15 day old great tits in response to a between-habitat cross-fostering manipulation between an urban and a rural population. a) Superoxide dismutase (SOD) activity in red blood cells, b) plasma total antioxidant capacity (AOX) corrected for uric acid; and c) oxidative damage measured as malondialdehyde (MDA) in plasma. For final models, model estimates for fixed effects and variance for random effects is showed, test statistic is *F* for fixed effects, χ^2^ for random effects. For dropped terms, log-likelihood ratio test valuess are shown together with p-values. In both cases denominator degrees of freedom obtained using the Satterwaite approximation. SE = standard error, d.f. = degrees of freedom, R_m_^2^= marginal R-squared, R_c_^2^= conditional R-squared.

1. *Superoxide dismutase (SOD): n = 139 nestlings; 16 cross-fostering pairs*

| term | estimate (SE) / variance | F / χ^2^ | d.f. | p-value |
| --- | --- | --- | --- | --- |
| Final model (R_m_^2^ = 0.033; R_c_^2^ = 0.78) |  |  |  |  |
| Intercept | 1.422 (0.167) |  |  |  |
| rearing habitat |  | 8.77 | 1, 18.33 | ***0.008*** |
| urban | 1.704 (0.173) |  |  |  |
| rural | 1.418 (0.167) | rural |  |  |
| *Random factors* |  |  |  |  |
| nest of rearing | 0.022 | 1.98 | 1 | 0.16 |
| nest of origin | 0.033 | 3.57 | 1 | 0.06 |
| assay plate | 0.413 | 116.01 | 1 | *2 x 10^-16^* |
| Residual | 0.122 |  |  |  |
| Dropped terms |  |  |  |  |
| habitat of origin |  | 3.02 | 1, 19.72 | 0.082 |
| body mass |  | 0.04 | 1, 108.55 | 0.832 |
| sex |  | 0.65 | 1, 106.80 | 0.421 |
| hatching date |  | 0.89 | 1, 34.38 | 0.344 |
| rearing habitat × habitat of origin  origin (urban) |  | 0.20 | 1, 112.15 | 0.656 |
| rearing habitat × body mass |  | 3.02 x 10^-3^ | 1, 114.34 | 0.956 |
| rearing habitat × sex |  | 7.57 x 10^-3^ | 1, 112.62 | 0.931 |
| rearing habitat × hatching date |  | 1.28 | 1, 23.47 | 0.257 |

1. *Total antioxidant capacity (AOX): n = 111 nestlings; 16 cross-fostering pairs*

| term | estimate (SE) / variance | *F* / *χ*^2^ | d.f. | *p*-value |
| --- | --- | --- | --- | --- |
| Final model (R_m_^2^ = 0.11; R_c_^2^ = 0.33) |  |  |  |  |
| Intercept | 0.884 (0.329) |  |  |  |
| hatching date | -0.005 (0.017) | 7.30 | 1, 22.81 | ***0.013*** |
| *Random factors* |  |  |  |  |
| nest of rearing | 0.004 | 4.28 | 1 | *0.04* |
| nest of origin | 3.29 x 10^-4^ | 3.23 x 10^-3^ | 1 | 0.86 |
| assay plate | 0.000^a^ | 2.39 x 10^-12^ | 1 | 1.00 |
| Residual | 0.014 |  |  |  |
| Dropped terms |  |  |  |  |
| habitat of origin |  | 0.52 | 1, 13.78 | 0.469 |
| rearing habitat |  | 0.94 | 1, 18.49 | 0.331 |
| body mass |  | 0.10 | 1, 100.97 | 0.754 |
| sex |  | 1.43 | 1, 102.11 | 0.231 |
| rearing habitat × habitat of origin |  | 0.13 | 1, 105.04 | 0.715 |
| rearing habitat × body mass |  | 0.08 | 1, 108.01 | 0.775 |
| rearing habitat × sex |  | 1.25 | 1, 106.56 | 0.264 |
| rearing habitat × hatching date |  | -8.50 x 10^-4^ | 1, 15.87 | 0.977 |

^a^ Parameter estimate was bound to 0

1. *Malondialdehyde (MDA): n = 71 nestlings; 16 cross-fostering pairs*

| term | estimate (SE) / variance | *F* / *χ*^2^ | d.f. | *p*-value |
| --- | --- | --- | --- | --- |
| Final model (R_m_^2^ = 0.22; R_c_^2^ = 0.29) |  |  |  |  |
| Intercept | 2.103 (1.236) |  |  |  |
| rearing habitat |  | 10.05 | 1, 34.44 | ***0.003*** |
| urban | 1.695 (0.070) |  |  |  |
| rural | 1.598 (0.053) |  |  |  |
| hatching date | 1.646 (0.044) | 6.93 | 1, 34.58 | ***0.013*** |
| rearing habitat × hatching date |  | 10.36 | 1, 34.58 | ***0.003*** |
| urban:hatching date | 1.695 (0.070) |  |  |  |
| rural:hatching date | 1.597 (0.053) |  |  |  |
| *Random factors* |  |  |  |  |
| nest of rearing | 0.008 | 6.55 x 10^-1^ | 1 | 0.40 |
| nest of origin | 0.000^a^ | 4.19 x 10^-13^ | 1 | 1.00 |
| Residual | 0.089 |  |  |  |
| Dropped terms |  |  |  |  |
| habitat of origin |  | 0.32 | 1, 61.56 | 0.569 |
| body mass |  | 1.24 | 1, 70.76 | 0.264 |
| sex |  | 0.10 | 1, 70.42 | 0.752 |
| rearing habitat × habitat of origin |  | 0.54 | 1, 64.68 | 0.460 |
| rearing habitat × body mass |  | 0.14 | 1, 69.04 | 0.712 |
| rearing habitat × sex |  | 0.26 | 1, 70.85 | 0.609 |

^a^ Parameter estimate was bound to 0

Table S2. Linear mixed models of three oxidative stress biomarkers in 15 day old great tits in response to within-habitat cross-fostering manipulation in an urban and a rural population (control experiment for the manipulation *per se*). a) Superoxide dismutase (SOD) activity in red blood cells, b) plasma total antioxidant capacity (AOX) corrected for uric acid; and c) oxidative damage measured as malondialdehyde (MDA) in plasma. Model estimates for fixed effects and variance for random effects are showed, test statistic is *F* for fixed effects, χ^2^ for random effects. In fixed factors denominator degrees of freedom are obtained using the Satterwaite approximation. SE = standard error, d.f. = degrees of freedom.

1. *Superoxide dismutase (SOD): n = 165 nestlings; 18 cross-fostering pairs ( 8 urban and 10 rural)*

| term | | estimate (SE) / variance | *F* / *χ*^2^ | d.f. | *p*-value |
| --- | --- | --- | --- | --- | --- |
| Intercept | | 5.97 (1.62) |  |  |  |
| rearing habitat | |  | 6.61 | 1, 34.05 | *0.010* |
|  | urban | 1.451 (0.147) |  |  |  |
|  | **rural** | 1.464 (0.143) |  |  |  |
| manipulation | |  | 0.16 | 1, 130.75 | 0.677 |
|  | cross-fostered | 1.468 (0.143) |  |  |  |
|  | non-cross-fostered | 1.447 (0.141) |  |  |  |
| body mass | | 1.457 (0.140) | 3 x 10^-3^ | 1, 147.58 | 0.976 |
| sex | |  | 3.25 | 1, 150.20 | 0.063 |
|  | male | 1.407 (0.142) |  |  |  |
|  | female | 1.509 (0.143) |  |  |  |
| hatching date | | 1.457 (0.140) | 4.72 | 1, 31.58 | *0.028* |
| rearing habitat × manipulation | |  | 0.80 | 1, 128.80 | 0.365 |
|  | urban:cross-fostered | 1.439 (0.155) |  |  |  |
|  | urban:non-cross-fostered | 1.464 (0.152) |  |  |  |
|  | rural:cross-fostered | 1.497 (0.146) |  |  |  |
|  | rural:non-cross-fostered | 1.431 (0.144) |  |  |  |
| rearing habitat × body mass | |  | 0.21 | 1, 147.51 | 0.653 |
|  | urban:body mass | 1.451 (0.147) |  |  |  |
|  | rural:body mass | 1.464 (0.142) |  |  |  |
| rearing habitat × sex | |  | 0.85 | 1, 149.06 | 0.331 |
|  | urban:male | 1.375 (0.154) |  |  |  |
|  | urban:female | 1.528 (0.145) |  |  |  |
|  | rural:male | 1.439 (0.145) |  |  |  |
|  | rural:female | 1.489 (0.145) |  |  |  |
| rearing habitat x hatching date | |  | 7.89 | 1, 29.69 | *0.006* |
|  | urban:hatching date | 1.451 (0.147) |  |  |  |
|  | rural:hatching date | 1.464 (0.142) |  |  |  |
| *Random factors* | |  |  |  |  |
| nest of rearing | | 0.018 | 9.95 | 1 | *0.003* |
| nest of origin | | 0.000^a^ | 3.13 x 10^-12^ | 1 | 1.00 |
| assay plate | | 0.323 | 1.64 x 10^2^ | 1 | *2 x 10^-16^* |
| Residual | | 0.008 |  |  |  |

^a^ Parameter estimate was bound to 0

1. *Antioxidant levels (AOX): n= 143 nestlings; 18 cross-fostering pairs (8 urban and 10 rural)*

| term | | estimate (SE) / variance | *F* / *χ*^2^ | d.f. | *p*-value |
| --- | --- | --- | --- | --- | --- |
| Intercept | | -0.17 (0.56) |  |  |  |
| rearing habitat | |  | 0.26 | 1, 28.51 | 0.612 |
|  | urban | -0.020 (0.020) |  |  |  |
|  | **rural** | 0.000 (0.016) |  |  |  |
| manipulation | |  | 0.71 | 1, 122.20 | 0.399 |
|  | cross-fostered | -0.017 (0.016) |  |  |  |
|  | non-cross-fostered | -0.003 (0.014) |  |  |  |
| body mass | | -0.010 (0.013) | 2.51 | 1, 141.96 | 0.115 |
| sex | |  | 0.03 | 1, 141.73 | 0.860 |
|  | male | -0.012 (0.015) |  |  |  |
|  | female | -0.008 (0.016) |  |  |  |
| hatching date | | -0.010 (0.013) | 0.09 | 1, 26.89 | 0.766 |
| rearing habitat × manipulation | |  | 0.14 | 1, 122.20 | 0.712 |
|  | urban:cross-fostered | -0.030 (0.026) |  |  |  |
|  | urban:non-cross-fostered | -0.010 (0.023) |  |  |  |
|  | rural:cross-fostered | -0.004 (0.019) |  |  |  |
|  | rural:non-cross-fostered | 0.004 (0.018) |  |  |  |
| rearing habitat × body mass | |  | 1.43 | 1, 141.96 | 0.233 |
|  | urban:body mass | -1.999 x 10^-2^ (0.020) |  |  |  |
|  | rural:body mass | -1.267 x 10^-5^ (0.016) |  |  |  |
| rearing habitat × sex | |  | 2.20 x 10^-5^ | 1, 141.73 | 0.988 |
|  | urban:male | -0.022 (0.025) |  |  |  |
|  | urban:female | -0.018 (0.025) |  |  |  |
|  | rural:male | -0.001 (0.018) |  |  |  |
|  | rural:female | 0.001 (0.018) |  |  |  |
| rearing habitat x hatching date | |  | 0.72 | 1, 26.89 | 0.402 |
|  | urban:hatching date | -1.999 x 10^-2^ (0.020) |  |  |  |
|  | rural:hatching date | -1.267 x 10^-5^ (0.016) |  |  |  |
| *Random factors* | |  |  |  |  |
| nest of rearing | | 0.002 | 1.03 x 10^1^ | 1 | *0.001* |
| nest of origin | | 0.000^a^ | 1.14 x 10^-13^ | 1 | 1.00 |
| assay plate | | 0.000^a^ | 1.14 x 10^-13^ | 1 | 1.00 |
| Residual | | 0.008 |  |  |  |

^a^ Parameter estimate was bound to 0

1. *Malondialdehyde (MDA): n= 73 nestlings; 18* *cross-fostering pairs (8 urban and 10 rural)*

| term | | estimate (SE) / variance | *F* / *χ*^2^ | d.f. | *p*-value |
| --- | --- | --- | --- | --- | --- |
| Intercept | | 1.95 (1.88) |  |  |  |
| rearing habitat | |  | 0.03 | 1, 27.79 | 0.857 |
|  | urban | 1.536 (0.083) |  |  |  |
|  | **rural** | 1.603 (0.057) |  |  |  |
| manipulation | |  | 1.35 | 1, 72.91 | 0.248 |
|  | cross-fostered | 1.514 (0.076) |  |  |  |
|  | non-cross-fostered | 1.625 (0.061) |  |  |  |
| body mass | | 1.570 (0.050) | 0.42 | 1, 73.00 | 0.520 |
| sex | |  | 1.46 | 1, 69.49 | 0.231 |
|  | male | 1.513 (0.070) |  |  |  |
|  | female | 1.627 (0.067) |  |  |  |
| hatching date | | 1.570 (0.050) | 0.10 | 1, 26.52 | 0.749 |
| rearing habitat × manipulation | |  | 1.10 | 1, 72.91 | 0.297 |
|  | urban:cross-fostered | 1.430 (0.124) |  |  |  |
|  | urban:non-cross-fostered | 1.643 (0.104) |  |  |  |
|  | rural:cross-fostered | 1.598 (0.089) |  |  |  |
|  | rural:non-cross-fostered | 1.609 (0.066) |  |  |  |
| rearing habitat × body mass | |  | 1.14 | 1, 73.00 | 0.289 |
|  | urban:body mass | 1.536 (0.083) |  |  |  |
|  | rural:body mass | 1.603 (0.057) |  |  |  |
| rearing habitat × sex | |  | 3.24 | 1, 69.49 | 0.076 |
|  | urban:male | 1.395 (0.123) |  |  |  |
|  | urban:female | 1.678 (0.106) |  |  |  |
|  | rural:male | 1.631 (0.073) |  |  |  |
|  | rural:female | 1.575 (0.083) |  |  |  |
| rearing habitat x hatching date | |  | 0.01 | 1, 26.52 | 0.902 |
|  | urban:hatching date | 1.536 (0.083) |  |  |  |
|  | rural:hatching date | 1.603 (0.057) |  |  |  |
| *Random factors* | |  |  |  |  |
| nest of rearing | | 0.002 | 2.92 x 10^-2^ | 1 | 0.90 |
| nest of origin | | 0.000^a^ | 6.39 x 10^-14^ | 1 | 1.00 |
| Residual | | 0.111 |  |  |  |

^a^ Parameter estimate was bound to 0
